# Supplementary material for: Density and population viability of coastal marten: a rare and geographically isolated small carnivore
Source: PeerJ. 2018 Apr 4;6:e4530. doi: 10.7717/peerj.4530 (PMC5889706; doi:10.7717/peerj.4530)
Supplement: Article S1 — We used JAGS (Plummer, 2003), accessed with the jagsUI package (Kellner, 2014) in R v. 3.2.3 (R Core Team, 2017), to run the spatial mark-resight analysis. Estimates were calculated from 3,000 MCMC samples, taken from 3 chains run for 10,000 iterations, thinned by five, following a burn-in of 5,000. Below is the code we used to fit our spatial mark-resight model with telemetry data to a population of coastal martens in central Oregon. Data files should be formatted in the structure below. [file peerj-06-4530-s001.docx]

**Supplementary Material – Article S1**

**Density and population viability of coastal marten: a rare and geographically isolated small carnivore**

**Mark A. Linnell**^1a^, **Katie M. Moriarty**^2a^, **David S. Green**^3^, **Taal Levi**^4^

**Bayesian modeling and example code**

We used JAGS (Plummer, 2003), accessed with the jagsUI package (Kellner, 2014) in R v. 3.2.3 (R Core Team 2016), to run the spatial mark-resight analysis. Estimates were calculated from 3,000 MCMC samples, taken from 3 chains run for 10,000 iterations, thinned by five, following a burn-in of 5,000. Below is the code we used to fit our spatial mark-resight model with telemetry data to a population of coastal martens in central Oregon. Data files should be formatted in the structure below.

model {

*## Data and definitions*

*# Baiting = days since last baiting at each camera location*

*# Sex = sex of each marten (0/1); males are 0, females are 1, NA for augmented individuals*

*# Y.trap = the number of times each marten was trapped i at each trapping location j*

*# Y.cam = whether or not (0/1) each marked marten i was seen at camera j on night k*

*# nU = the total number of unmarked martens seen at camera j on night k*

*# locs = X/Y coordinates of each telemetry location for collared individuals*

*# off = matrix indicating which rows of telemetry locations are assigned to each individual*

*# K.trap = the number of nights each trap was open during the trapping season*

*# K.cam = the number of nights the cameras were deployed*

*# n.cams = the number of cameras deployed in the resight transect*

*# N.marked = the number of individuals that were marked (captured)*

*# nG = number of grid cells in the state space*

*# M = number of individuals to monitor in the augmented dataset*

*# s = activity center*

*# S = X/Y coordinates of each grid centroid*

*# x0g = x coordinate of grid cell center*

*# y0g = y coordinate of grid cell center*

*# nU = the number of unmarked individuals seen on camera j on day k*

*# pixArea = area of the pixels*

*# n.collar = number of collared animals*

*# n.traps = number of trapping locations*

*# x0g = x coordinate of activity center*

*# y0g = y coordinate of activity center*

*# cam.x = X coordinate of each camera*

*# cam.y = Y coordinate of each camera*

*# trap.x = X coordinate of each trap*

*# trap.y = Y coordinate of each trap*

# Priors and constraints

psi.sex ~ dunif(0,1) # Prior for sex

alpha0 ~ dunif(-5,5) # Intercept of canopy cover

alpha1 ~ dnorm(0,1) # Effect of canopy cover

beta0 ~ dunif(-5,5) # Intercept for capture probability

beta1 ~ dnorm(0,1) # Effect of sex on capture probability

delta0 ~ dunif(-5,5) # Intercept for resight probability

delta1 ~ dnorm(0,1) # Effect of sex on resight probability

delta2 ~ dnorm(0,1) # Effect of days-since-baiting on resight probability

for(s in 1:2){

sigma[s] ~ dunif(0,5000) # Movement parameter by sex

sigma2[s] <- sigma[s]^2

}

# Intensity function for discrete state-space habitat covariates

for(g in 1:nG){

mu[g] <- exp(alpha0 + alpha1*((cancov[g]-mean(cancov[]))/sd(cancov[])))*pixArea

probs[g] <- mu[g]/EN # Probability of an activity center being in a grid cell

} #g

EN <- sum(mu[])

psi <- EN/(M)

# Likelihoods

for(i in 1:M){

z[i] ~ dbern(psi) # Inclusion probability

Sex[i] ~ dbern(psi.sex) # Probability of sex

Sex2[i] <- Sex[i] + 1

s[i] ~ dcat(probs[]) # activity center

x0g[i] <- S[s[i],1]

y0g[i] <- S[s[i],2]

for(j in 1:n.cams){

for(k in 1:K.cam){

logit(lam0.resight[i,j,k]) <- delta0 + delta1*Sex[i] + delta2*Baiting[j,k]

}

}

logit(lam0.mark[i]) <- beta0 + beta1*Sex[i]

for(j in 1:n.traps) { # Likelihood for marking observations (captures)

d.mark2[i,j] <- (x0g[i]-trap.x[j])^2 + (y0g[i]-trap.y[j])^2

p.mark[i,j] <- lam0.mark[i]*exp(-d.mark2[i,j]/(2*sigma2[Sex2[i]]))*z[i]

Y.trap[i,j] ~ dbinom(p.mark[i,j], K.trap[j])

}

for(j in 1:n.cams){ # Resight probability

d.resight2[i,j] <- (x0g[i]-cam.x[j])^2 + (y0g[i]-cam.y[j])^2

}

}

# Camera data

for (i in 1:n.marked){

for (j in 1:n.cams){

for (k in 1:K.cam){

Y.cam[i,j,k] ~ dbern(p.resight[i,j,k])

}

}

}

# Unknown individuals

for (i in 1:M){

for (j in 1:n.cams){

for (k in 1:K.cam){

yu[i,j,k] ~ dbern(p.resight[i,j,k]*(1 - marked[i]))

p.resight[i,j,k] <- lam0.resight[i,j,k]*exp(-d.resight2[i,j]/(2*sigma2[Sex2[i]]))*z[i]

}

}

}

# Likelihood for unmarked animals

for (j in 1:n.cams){

for (k in 1:K.cam){

nU[j,k] ~ dsum(yu[1,j,k], yu[2,j,k], yu[3,j,k], yu[4,j,k], yu[5,j,k], yu[6,j,k], yu[7,j,k], yu[8,j,k],

yu[9,j,k], yu[10,j,k], yu[11,j,k], yu[12,j,k], yu[13,j,k], yu[14,j,k], yu[15,j,k], yu[16,j,k], yu[17,j,k],

yu[18,j,k], yu[19,j,k], yu[20,j,k], yu[21,j,k], yu[22,j,k], yu[23,j,k], yu[24,j,k], yu[25,j,k], yu[26,j,k],

yu[27,j,k], yu[28,j,k], yu[29,j,k], yu[30,j,k], yu[31,j,k], yu[32,j,k], yu[33,j,k], yu[34,j,k], yu[35,j,k])

}

}

# Telemetry data for collared animals

for (i in 1:n.collar){

for (r in off1[i]:off2[i]){

locs[r,1] ~ dnorm(x0g[i], 1/(sigma[Sex2[i]]^2))

locs[r,2] ~ dnorm(y0g[i], 1/(sigma[Sex2[i]]^2))

}

}

}
