# Supplementary material for: Density and population viability of coastal marten: a rare and geographically isolated small carnivore
Source: PeerJ. 2018 Apr 4;6:e4530. doi: 10.7717/peerj.4530 (PMC5889706; doi:10.7717/peerj.4530)
Supplement: Table S1 — Summary of location data collected by individual Pacific marten (Martes caurina). We collected spatial locations (Lxns) on marten when they were radio collared (Tracking period) with either a G10 snap technology GPS unit (27g, Advanced Telemetry Systems, “ATS”), a M1820 VHF unit made by ATS (27 g; “VHF”), or a Quantum 4,000 micro-mini GPS unit made by Telemetry Solutions (41–44 g, “TS”). Location data were collected in the Oregon Dunes Recreation Area between October 2015 and January 2016. We estimated territories using 99% Local Convex Hulls (LoCoH). [file peerj-06-4530-s002.docx]

**Supplemental information to submitted article: Density and population viability of coastal marten: a rare and geographically isolated small carnivore**

**Mark A. Linnell**^1a^, **Katie M. Moriarty**^2a^, **David S. Green**^3^, **Taal Levi**^4^

Table 1a. Summary of location data collected by individual Pacific marten (*Martes caurina*). We collected spatial locations (Lxns) on marten when they were radio collared (Tracking period) with either a G10 snap technology GPS unit (27g, Advanced Telemetry Systems, “ATS”), a M1820 VHF unit made by ATS (27g; “VHF”), or a Quantum 4000 micro-mini GPS unit made by Telemetry Solutions (41-44g, “TS”). Location data were collected in the Oregon Dunes Recreation Area between October 2015 and January 2016. We estimated territories using 99% Local Convex Hulls (LoCoH) and here we report distance from territory center to the furthest edge of their territory (Radius).

| Marten  (ID) | Tracking period*^a^*  (dates) | Lxns  (#) | Collar  (brand) | 99% LoCoh (units) | Length (m) | Forest cover*^b^* (km^2^) |
| --- | --- | --- | --- | --- | --- | --- |
| F01*^c^* | 11/22/15 - 01/05/16 | 2623 | ATS | 0.59 | 0.72 | 0.42 |
| F03*^c^* | 10/14/15 - 10/29/15 | 752 | TS | 0.62 | 0.77 | 0.49 |
| F04 | 10/14/15 - 11/25/15 | 37 | VHF | 0.79 | 1.50 | 0.52 |
| F05 | 10/18/15 - 01/19/16 | 33 | VHF | 0.71 | 0.90 | 0.53 |
| F06*^c^* | 10/29/15 - 12/29/15 | 23 | VHF | 0.27*^d^* | 6.60 | - |
| F07*^c^* | 10/29/15 - 01/27/15 | 35 | VHF | 0.84 | 1.02 | 0.68 |
| F08*^c^* | 11/29/15 - 12/24/15 | 2960 | ATS | 0.64 | 7.60 | 0.41 |
| M01 | 12/25/15 - 01/01/16 | 666 | ATS | 2.2 | 1.45 | 0.75 |
| M02*^c^* | 10/16/15 - 10/26/15 | 477 | TS | 2.2 | 1.85 | 0.50 |
| M03*^c^* | 11/30/15 - 12/05/15 | 324 | TS | 1.7 | 1.17 | 1.19 |
| M04 | 11/23/15 - 11/26/15 | 173 | TS | 1 | 1.20 | 0.78 |

*^a^*For GPS collars (ATS, TS), animals were also tracked using VHF until the end of the study.

*^b^*Forest cover (>40% forest cover within 100m moving window) within 99% LoCoh territory.

*^c^*Located within spatial mark-recapture transect in the northern subpopulation.

*^d^*Not used in territory size estimation summary due to malfunction of VHF which limited signal strength and therefore estimation of space use.
