# Supplementary material for: Density and population viability of coastal marten: a rare and geographically isolated small carnivore
Source: PeerJ. 2018 Apr 4;6:e4530. doi: 10.7717/peerj.4530 (PMC5889706; doi:10.7717/peerj.4530)
Supplement: Table S2 — Technical articles that included the keywords “marten”, “density”, “territory”, and “home range”. Of the >75 papers reviewed for North American martens (Martes americana, M. caurina), four reported both home range sizes and density. Territories were estimated using either 100% Minimum Convex Polygons (MCP) or time-influenced Local Convex Hulls (t-LoCoH). Reported densities were either minimum known alive (MNKA) or calculated with spatial mark-recapture (SMR). Studies were conducted in Maine, USA (Payer & Harrison, 1999), central British Columbia, Canada (Poole et al., 2004), New Hampshire, USA (Sirén et al., 2016), and Quebec, Canada (Godbout & Ouellet, 2010). [file peerj-06-4530-s003.docx]

**Supplemental information to submitted article: Density and population viability of coastal marten: a rare and geographically isolated small carnivore**

**Mark A. Linnell**^1a^, **Katie M. Moriarty**^2a^, **David S. Green**^3^, **Taal Levi**^4^

Table 2. Technical articles that included the keywords “marten”, “density”, “territory”, and “home range”. Of the >75 papers reviewed for North American martens (*Martes americana, M. caurina*), four reported both home range sizes and density. Territories were estimated using either 100% Minimum Convex Polygons (MCP) or time-influenced Local Convex Hulls (t-LoCoH). Reported densities were either minimum known alive (MNKA) or calculated with spatial mark-recapture (SMR). Studies were conducted in Maine, USA (Payer and Harrison 1999), central British Columbia, Canada (Poole et al. 2004), New Hampshire, USA (Siren et al. 2016), and Quebec, Canada (Godbout and Ouellet 2010).

|  | Territory size estimates (km^2^ ± *SD*, *n*) | | |  | Density (*n*/km2) | |  |
| --- | --- | --- | --- | --- | --- | --- | --- |
| Study | Male | Female | Range Estimator |  | Density | Density Estimator |  |
| Payer and Harrison 1999^a^ | 3.25 ± 1.42, 41 | 1.65 ± 0.86, 12 | 100% MCP |  | 0.47 | MNKA |  |
| Payer and Harrison 1999^b^ | 4.44 ± 2.07, 25 | 2.38 ± 1.35, 19 | 100% MCP |  | 0.11 | MNKA |  |
| Payer and Harrison 1999^c^ | 4.89 ± 2.23, 39 | 2.88 ± 1.15, 39 | 100% MCP |  | 0.17 | MNKA |  |
| Poole et al. 2004 | 3.30 ± 0.42, 22 | 2.00 ± 0.4, 7 | 100% MCP |  | 0.34 | MNKA |  |
| Siren et al. 2016 | 2.95 ± 0.27, 11 | 1.55 ± 0.2, 4 | 100% MCP |  | 0.51±0.16 | SMR |  |
| Godbout and Ouellet 2010 | 5.50 ± 1.0, 6 | 3.9 ± n/r, 6 | 100% MCP |  | 0.22±0.002 | –^d^ |  |
| This study | 1.80 ± 0.6, 4 | 0.7 ± 0.1, 6 | 99% LoCoH |  | 1.01±0.1 | SMR |  |

^a^Forest reserve

^b^Trapped industrial

^c^Untrapped industrial

^d^Estimator was unreported
