# Supplementary figures and images for: Density and population viability of coastal marten: a rare and geographically isolated small carnivore
Source: PeerJ. 2018 Apr 4;6:e4530. doi: 10.7717/peerj.4530 (PMC5889706; doi:10.7717/peerj.4530)

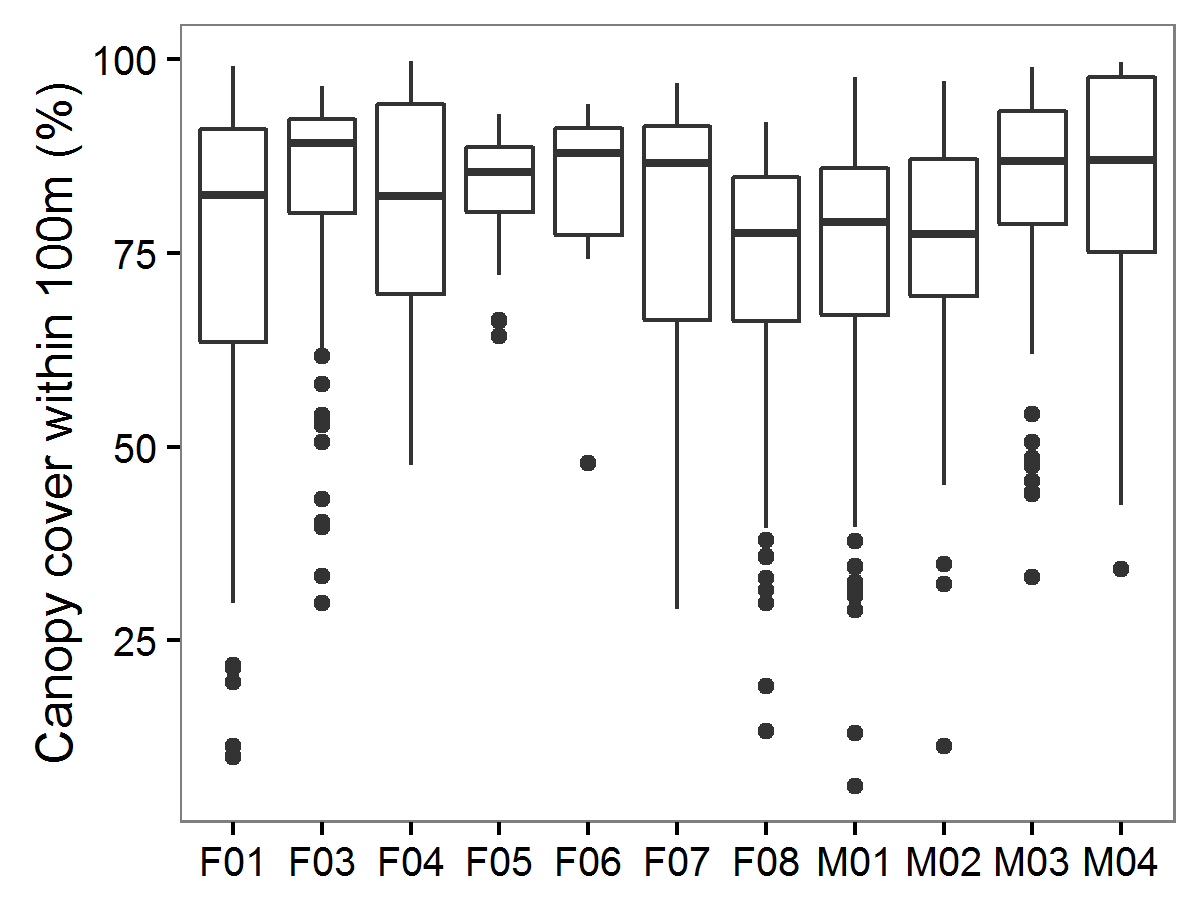

Supplement: Figure S1 — Percent forest cover (vegetation height >1 m estimated from LiDAR) within a 100 m moving radius of spatial locations collected using VHF and GPS telemetry of martens in coastal Oregon from October 2015 to January 2016. The average lower quantile combined with the demonstrated forested amount within home ranges were used to inform thresholds for territory mapping. [file peerj-06-4530-s004.png]
